# Fe and Zn stress induced gene expression analysis unraveled mechanisms of mineral homeostasis in common bean (*Phaseolus vulgaris* L.)

**DOI:** 10.1038/s41598-021-03506-2

**Published:** 2021-12-15

**Authors:** Uneeb Urwat, Syed Mudasir Ahmad, Antonio Masi, Nazir Ahmad Ganai, Imtiyaz Murtaza, Imran Khan, Sajad Majeed Zargar

**Affiliations:** 1 Proteomics Laboratory, Division of Plant Biotechnology, Sher-E-Kashmir University of Agricultural Sciences & Technology of Kashmir, Shalimar, Srinagar, Jammu & Kashmir, India; 2 Division of Animal Biotechnology, Sher-E-Kashmir University of Agricultural Sciences & Technology of Kashmir, Shuhama Campus, Jammu & Kashmir, India; 3 Department of Agronomy, Food, Natural Resources, Animals and Environment (DAFNAE), University of Padova, Padova, Italy; 4 Divisions of Basic Sciences, Sher-E-Kashmir University of Agricultural Sciences & Technology of Kashmir, Shuhama Campus, Jammu & Kashmir, India; 5 Division of Statistics, Sher-E-Kashmir University of Agricultural Sciences & Technology of Kashmir, Shuhama Campus, Jammu & Kashmir, India

**Keywords:** Biotechnology, Plant sciences

## Abstract

Iron (Fe) and zinc (Zn) stress significantly affects fundamental metabolic and physiological processes in plants that results in reduction of plant growth and development. In the present study, common bean variety; Shalimar French Bean-1 (SFB-1) was used as an experimental material. Four different MGRL media i.e. normal MGRL medium (Control), media without Fe (0-Fe), media without Zn (0-Zn) and media with excess Zn (300-Zn) were used for growing seeds of SFB-1 under in vitro condition for three weeks under optimum conditions. Three week old shoot and root tissues were harvested from the plants grown in these four different in vitro conditions and were, subjected to Fe and Zn estimation. Further, extraction of total RNA for differential gene expression of ten candidate genes selected based on our in silico investigation and their classification, phylogeny and expression pattern was unraveled. Expression analysis of three candidate genes (OPT3, NRAMP2 and NRAMP3) in roots revealed possible cross talk among Fe/Zn stress that was further confirmed by observing less accumulation of Fe in roots under both these conditions. However, we observed, higher accumulation of Fe in shoots under 0-Fe condition compared to control that suggests precise sensing for priority based compartmentalization and partitioning leading to higher accumulation of Fe in shoots. Furthermore, the expression analysis of IRT1, FRO1 and Ferritin 1 genes under Fe/Zn stress suggested their role in uptake/transport and signaling of Fe and Zn, whereas the expression of ZIP2, NRAMP1, HA2 and GLP1 genes were highly responsive to Zn in *Phaseolus vulgaris*. The identified genes highly responsive to Fe and Zn stress condition can be potential candidates for overcoming mineral stress in dicot crop plants.

## Introduction

Mineral uptake and assimilation in plants are directly influenced by changes in environmental conditions^1^. Most common type of mineral deficiencies is of iron (Fe), zinc (Zn) and calcium (Ca), while other deficiencies are believed to be rare^2^. These micronutrients (Fe and Zn) are essential for regulation of many cellular processes in plants like chlorophyll synthesis, photosynthesis and respiration, transcription and their deficiencies leads to impaired cellular metabolism which in turn severely affects plant growth and development^3^. The response of plants to mineral stress involves a complex regulatory cascade and activates number of defense mechanisms to enhance stress tolerance that ultimately result in changing patterns of gene expression, protein synthesis, primary and secondary metabolism, imbalance in uptake, distribution, storage^3^ or even compartmentalization and partitioning of these minerals^4^ and carbohydrates^5^. The capacity for compartmentalization and partitioning of minerals and carbohydrates among different tissues and cells is not only the driving force for the development of different structures^6^ and biofortify edible parts but also the key mechanism regulating mineral stress tolerance in plants^7^. To ensure optimal metabolic rates, plants regulate changes in expression of key transporters and metal homeostasis proteins that play important role in compartmentalization and partitioning of these minerals in different cellular organelles and tissues^4^.

Common bean is a nutritional crop, a rich source of proteins, minerals and can be a good dicot crop model plant to understand the mechanisms underlying the adaptation of common bean to low nutrient input^8^. Differentially expressed genes in common bean grown under different mineral treatments shall indicate its defense mechanism upon mineral stress and provide initial breakthrough of genes possibly involved in recognition of events and early signaling responses to combat the mineral stress. Thus, gene expression analysis in common bean under mineral stress is necessary to decipher the complex molecular mechanisms involved in mineral stress tolerance.

Here we started with in silico based identification and characterization of potential Fe/Zn responsive candidate genes. Ten Fe/Zn responsive genes were identified in uncharacterized genome of common bean, and their classification, phylogeny and expression pattern was unraveled. As such our investigation provided the basis to reveal the molecular regulation of Fe/Zn responsive genes in case of a dicot legume crop model common bean. Further, we conducted differential gene expression of these ten candidate genes in three weeks old common bean (SFB-1) tissues grown under different mineral treatments. The outcome of differential gene expression studies when correlated with biochemical parameters provided some exciting results. This study provided new insight about the cross talk under Fe deficiency and excess Zn (that may be responsible for Fe deficiency) stress in common bean roots based on differential expression levels of candidate genes (OPT3, NRAMP2 and NRAMP3) that also correlated with the less accumulation of Fe in roots under these conditions, although a detailed analysis is needed to fully understand the regulatory network involved in this cross talk. We also observed higher accumulation of Fe in shoots under 0-Fe condition (unexpected) compared to control. However, the total concentration of Fe in plant under 0-Fe was less compared to the concentration of Fe in plants grown under control condition. This partitioning leads to higher accumulation of Fe and Zn in shoots as compared to roots of common bean and suggests that common bean plants prioritize partitioning of minerals towards the tissues as an adaptive mechanism during early stages of mineral stress. The present study also suggested the role of IRT1, FRO1 and Ferritin 1 gene under Fe/Zn stress in uptake/transport and signaling of Fe and Zn stress but the expression pattern of other three candidate genes/transporters-ZIP2, NRAMP1 and HA2 suggested their role in transportation/uptake of Zn whereas GLP1 reveals its role to overcome heavy metal stress in *Phaseolus vulgaris*. The genes highly responsive to Fe and Zn stress condition, suggest possible mechanisms of mineral homeostasis in common bean.

## Material and Methods

### In silico identification of Fe and Zn responsive genes/transporters in Phaseolus vulgaris

To conduct the in silico identification of ten Fe/Zn responsive proteins present in *Phaseolus vulgaris* proteome, the nucleotide and amino acid sequences of the Fe/Zn responsive genes of dicot plants like *Arabidopsis thaliana*, *Glycine max*, *Medicago truncatula* and monocots like *O. sativa*, *Triticum aestivum, Hordeum vulgare* and others available in public domain/literature, were collected from the National Center for Biotechnology Information (NCBI) website (http://www.ncbi.nlm.nih.gov/). These retrieved sequences were used as queries in basic local alignment search tool (BLASTp) against *P. vulgaris* and other dicot plants in Phytozome (https://phytozome.jgi.doe.go). All sequences with similarity score above 80% and e-value 0.01 were considered. All retrieved hits were examined by using hidden Markov models (HMMs) provided in Pfam 32.0 (http://pfam.xfam.org/search) and other biological databases were used for confirmation of these identified sequences like PANTHER, InterPro and Gene Ontology.

### Prediction of physicochemical characteristics, subcellular localization, and transmembrane helixs (TMHs)

The protein molecular weights and pIs were predicted using ProtParam tool of ExPASy (http://web.expasy.org/protparam/)^9^. The probable subcellular localization was determined by CELLO online tool v.2.5 (http://cello.life.nctu.edu.tw/)^10^, Target P^11^, plant-mLoc^12^ (www.csbio.sjtu.edu.cn) and WoLF PSORT^13^. The transmembrane helices of identified proteins were predicted by using TMHMM server 2.0 (http://www.cbs.dtu.dk/services/TMHMM/).

### Gene structure visualization and conserved motif identification in Fe/Zn responsive genes

The exon–intron number and gene architecture of the identified Fe/Zn responsive genes in *Phaseolus vulgaris* were obtained using Gene Structure Display Server 2.0 (GSDS; http://gsds.cbi.pku.edu.cn/) by aligning the FASTA formatted CDS and genomic DNA sequences^14^. MEME analysis (http://meme-suite.org/) was performed with default parameters to identify conserved motifs in the identified Fe/Zn responsive protein sequences.

### Phylogenetic analysis

The evolutionary relationship of Fe/Zn responsive proteins of *Phaseolus vulgaris* with other dicot plant species such as *A. thaliana, P. vulgaris, G.max, F. vesca, P. persica, M. trancatula, T. pratense, M. domestica, C. sativus, A. halleri, A. lyrata, B. stricta, E. salsugineum, B. capitata, C. grandiflora, G. raimondii, T. cacao, C. rubella, C. papaya, B. rapa, C. elementina and C. sinensis* and monocots (O*ryza sativa, Hordeum vulgare* and *Zea mays*) was studied using MEGA 7 version 7.0 (https://www.megasoftware.net)^15^. Phylogenetic analysis was performed by maximum likelihood method in MEGA 7. Bootstrap analysis was performed with 1000 replicates. A combined multigene (10 identified Fe/Zn responsive proteins) phylogenetic tree was generated and visualized in FigTreev1.4.2.

### Physical mapping of Fe/Zn responsive genes in *Phaseolus vulgaris*

MapInspect 1.0 (https://mapinspect.software.informer.com) was used to physically map the genes onto individual chromosomes of common bean.

### Protein Interaction Network analysis

The Fe/Zn responsive protein interaction network was examined using the STRING online server (https://string-db.org/cgi accessed) and network generated was formatted in Cytoscape 3.8.2.

### In vitro growth of *Phaseolus vulgaris* L. plants

In the present study the common bean variety SFB-1 (Shalimar French Bean-1) used, was collected by fulfilling the institutional/national/international guidelines and legislation. This variety is released by Sher-e-Kashmir University of Agricultural Sciences and Technology of Kashmir- Shalimar, Srinagar, which is our parent University. The seed material was procured from Division of Genetics and Plant Breeding and the material was identified by Sajad M Zargar. The seeds of this variety are available in SKUAST-K and can be used by farmers for cultivation or by the researchers for research purpose. Seeds of Shalimar French Bean-1 (SFB-1) variety were surface-sterilized and were germinated on 4 different sterile MGRL media i.e. normal MGRL medium (Control), without Fe (0-Fe), without Zinc (0-Zn) and with 300 μM ZnSO_4_ (300-Zn). For in vitro growth of plants we have used our standardized method as detailed in Urwat et al.^16^. After 3 weeks growth, Fe and Zn was estimated and total RNA was extracted from the shoots and roots of the plants grown under all 4 conditions (normal, 0-Fe, 0-Zn and Excess Zn) as detailed above.

### Estimation of Fe and Zn content

From 3 weeks old plants grown under different mineral conditions (as detailed above), shoots and roots were harvested and were dried separately and crushed to fine powder. Further, Fe and Zn contents from all replicated samples were determined using Inductively Coupled Plasma Mass Spectrometry (ICP-MS).

### RNA extraction and quantification

Total RNA was extracted from both shoot and root tissues of SFB-1 grown in vitro under 4 different conditions as detailed above, using Trizol method as per the manufacturer’s instructions^16^. The quantity and quality of isolated RNA was checked at 260 and 280 nm with Nanodroplite (ThermoScientific) and UV–visible spectrophotometer (Thermo Scientific). Prior to reverse transcription, isolated total RNA samples were run on a 1% agarose gel. To rule out DNA contamination DNase treatment was given by using DNase kit (Sigma Aldrich, USA)^16^.

### cDNA synthesis and amplification of genes

cDNA synthesis was performed with equal concentration of RNA (1 μg) in all the samples using Thermo Scientific Revert Aid First Strand cDNA Synthesis Kit using oligo dT primers as per manufacturer instruction. The integrity of cDNA synthesis was checked by conventional PCR using cDNA as template and 2 house keeping genes (actin and tubulin) as primers that amplify 175 bp and 120 bp gene fragments respectively. For the validation of genes, cDNA as template and the forward and reverse primers of nine target genes given in Table 1 were used to amplify by PCR. The amplified PCR products were electrophoretically separated on 2% agarose gel. Details of primer sequences and amplicon size of all genes are given in Table 1. Reported primers were used for Actin^17^ and Ferritin gene^18^ and primers used for the rest of the genes were designed by using PRIMER 3 Plus program. The amplicons were sequenced and submitted to NCBI and their assigned accession no. are given in Table 1.

**Table 1:** List of genes and their primer sequences.

S.No	GENE	Primer sequence (5′ → 3′)	Tm $^\circ$C	Amplicon Size	Gene Accession No	Reference
1.	FER1	AAGCAGGAACCTTGGTGTTTCT CCTCCTCAAAGGGTTCAAAGATCAC	64.9 $^\circ$C 68.6$^\circ$C	73 bp	KF033276.1	Reported (Ayala-Vela et al.,2008)
**2.**	FRO1	TGGTGAGTGGTGGAAGTGG TGAGCCAGAAACAGAAAGCA	65.0$^\circ$C 63.7$^\circ$C	164 bp	MT921014	This study
3	GLP1	CCGAGATTGTGTTTGTGCTG ATGGACTGTGTGCCAGGTAA	57.08 $^\circ$C 57.78 $^\circ$C	199 bp	MW848439	This study
4	HA2	CTTTATTGAACGCCCTGGAT CAACCCCAACCAACTCCTTT	52.8$^\circ$C 55.6$^\circ$C	123 bp	MW603452	This study
5	IRT1	TGCTCAACGCTTCTTCTGCT CACCCGCACCTAAAAACACT	57.2$^\circ$C 55.7$^\circ$C	141 bp	MT921015	This study
6	NRAMP1	CTCATTCCCTTGCTTTGCTT GCCGTTTATCACTATCACCAGA	57.2 °C 57.2 °C	117 bp	MT928298	This study
7	NRAMP2	TGACCGCAGCAAGAAAGG TATCACCAGAGCAGCCACGA	65.3 °C 67.0 °C	568 bp	MT921011	This study
8	NRAMP3	GCGTTGATTACCCGAAGTTG GAACATTGAGCCACCCATTC	54.2 °C 54.1 °C	106 bp	MK911718	This study
9	OPT3	TTCGTCTTCCGCTACAACAA CATTTCACTTCCCCACCATT	54.2 °C 53.1 °C	150 bp	MT921013	This study
10	ZIP2	ACAAACTTCCCTGTGAGTGTTC TGTGACGACCAAATCCATAA	62.0 °C 61.4 °C	130 bp	MT921012	This study
11	Actin*	GAAGTTCTCTTCCAACCATCC TTTCCTTGCTCATTCTGTCCG	61.5 °C 66.3 °C	175 bp	KF033666	Reported Chen et al. 2009
12	Tubulin*	TCGTGGGTTTCAGCAGTAT GCAAAGGCAGTCAAGTATCG	56.71 °C 55.96 °C	120 bp	MT292610	This study

* Housekeeping genes.

### Relative quantification by qRT-PCR

Total RNA was extracted from both shoot and root tissues of plants grown under 4 different conditions (control, 0-Fe, 0-Zn, 300Zn). cDNA was synthesized as mentioned above. Actin and Tubulin was used as internal control for normalization of all the reactions. Primers used for normalization (ACT-F, ACT-R and TUB-F, TUB-R) and expression analysis of ten target genes (IRT1F- IRT1R, FER1F-FER1R, ZIP2F-ZIP2R, GLP1F-GLP1R, FRO1F-FRO1R, OPT3F-OPT3R, NRAMP1F-NRAMP1R, NRAMP2F-NRAMP2R, NRAMP3F-NRAMP3R, AHA2F-AHA2R and GLP1F-GLP1R) are given in Table 1. Criteria for selection of genes were based on their major role/involvement of genes for transport and accumulation followed in Strategy I by dicot plants^19^. qRT-PCR reactions were performed on a Light Cycler 480 (Roche) using KAPA SYBR® FAST qPCR Master Mix kit as per manufactures instruction. Amplicon dissociation curve was also recorded at the end of the PCR cycles. All reactions were run in triplicate and repeated twice. Relative expression of genes was analyzed by using Livak and Schmittgen^20^ method.

### Statistical analysis

The results obtained are the mean values of 12 and 6 biological replicates for Fe and Zn estimation and expression analysis respectively each with three technical replicates for all four treatments. The data was analyzed using one-way analysis of variance (ANOVA) followed by post hoc test (Multiple Comparisons) using SAS software (statistical analysis software institute, Cary, NC, USA) to investigate significant differences among multiple samples at 0.05 levels^16^.

## Results

### Identification and annotation of Fe/Zn responsive genes

A total of 10 Fe/Zn responsive genes/proteins were identified in *Phaseolus vulgaris* genome through blastp search by using sequences available in the NCBI as queries in Phytozome. These retrieved sequences were then examined by other databases to examine their protein domain for identifying their family /superfamily. Databases like pfam, InterPro, PANTHER and Gene Ontology were employed to identify the family /superfamily to which the predicted Fe/Zn responsive proteins belong and their profile IDs under which they are classified into family /superfamily are given in (Supplementary Table1). The protein characteristics such as coding sequence (CDS) lengths, protein lengths, isoelectric point, and molecular weight of identified Fe/Zn responsive proteins in *Phaseolus vulgaris* were evaluated, listed in Table 2. The sub-cellular localization of these 10 Fe/Zn responsive proteins was predicted using four different prediction tools. In most of the Fe/Zn responsive proteins, the results were the same with all the four tools. The determination of the subcellular localization of Fe/Zn responsive proteins will help understand the molecular function. Details of same are provided in Table 2.

**Table 2:** Summarized information of identified Fe/Zn responsive genes /protein features in *Phaseolus vulgaris L.*

S.No	Genes identified	Gene ID	Gen Length (bp)	CDS length (bp)	Intron Number	Exon Number	Protein length (aa)	Molecular Weight (KDa)	pI	No. of predicted TMHs	Sub-cellular localization
1	PvFER1	Phvul.008G093700	2809	765	7	8	254	28.30	5.64	0–1	Choloroplast
2	PvFRO1	Phvul.007G073900	5217	2037	7	8	678	77.37	9.1	8	Plasma membrane
3	PvGLP1	Phvul.001G221200	1624	654	1	2	217	22.95	8.85	0	Cell wall/extracellular
4	PvHA2	Phvul.009G239000	8327	2865	15	16	955	104.95	6.56	8	Plasmamembrane
5	PvIRT1	Phvul.002G099700	2566	1074	2	3	357	38.55	8.13	8	Plasmamembrane
6	PvNRAMP1	Phvul.005G182000	6104	1635	12	13	544	58.99	8.74	12	Plasma membrane
7	PvNRAMP2	Phvul.003G238600	3823	1551	3	4	516	56.68	5.36	10	Plasma membrane
8	PvNRAMP3	Phvul.002G014300	3638	1524	3	4	507	55.67	5.15	10	Plasma membrane
9	PvOPT3	Phvul.003G086500	4439	2226	5	4	741	83.27	9.04	16	Plasma membrane
10	PvZIP2	Phvul.010G059200	3958	1770	4	5	589	61.32	8.89	12	Plasma membrane

ID: identity; bp: base pair; aa: amino acids; pI: isoelectric point; MW: molecular weight; KDa: Kilo dalton.

### Phylogenetic analysis

**Figure 1:**
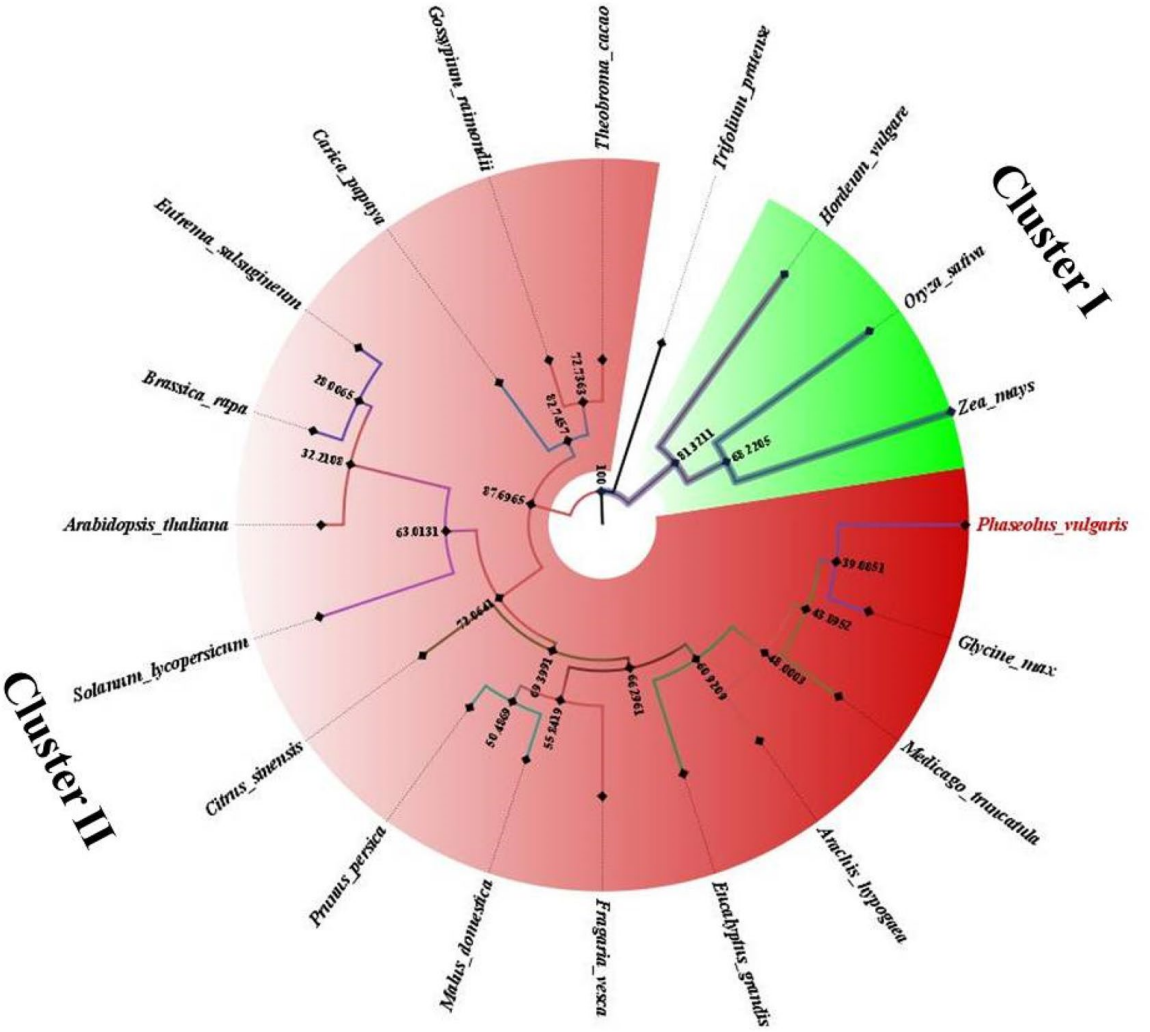
Combined multigene phylogenetic tree of Fe/Zn responsive proteins in 20 plant species. The circular phylogenetic tree was constructed by MEGA 7.0 software with the ML method for 1000 replicates bootstrap and visualized by FigTree v1.4.2. Red and green colors represent cluster I & II respectively.

To unravel the phylogenetic relationships and functional divergence of Fe/Zn responsive genes in *Phaseolus vulgaris* with Fe/Zn responsive homologs from other dicot plants and three monocots (used as outgroup) like *Oryza sativa*, *Hordeum vulgare* and *Zea mays*, the multiple sequence alignments and phylogenetic relationship analysis was carried out (Fig. 1). An unrooted maximum-likelihood phylogenetic tree based on Poisson correction model with the amino acid sequences of 20 plants using MEGA 7.0 software was constructed with default parameters and the reliability of interior branches was assessed with 1000 bootstrap repetitions (BT values ≥ 70%) and final tree was visualized in Figtree v1.4.2. The tree constructed was combined multigene phylogenetic tree of 10 identified Fe/Zn responsive proteins from 20 plant species. The tree was divided into two main clusters—cluster I and cluster II. Cluster I (green) included sequences of monocot plant species and cluster II (red) contained Fe/Zn responsive homologs of dicot plants. A clear monocot/dicot separation was observed in the phylogenetic tree. This tree also indicates *Phaseolus vulgaris* and *Glycine max* as very similar to each other and closely related (Fig. 1).

### Conserved motif analysis

**Figure 2:**
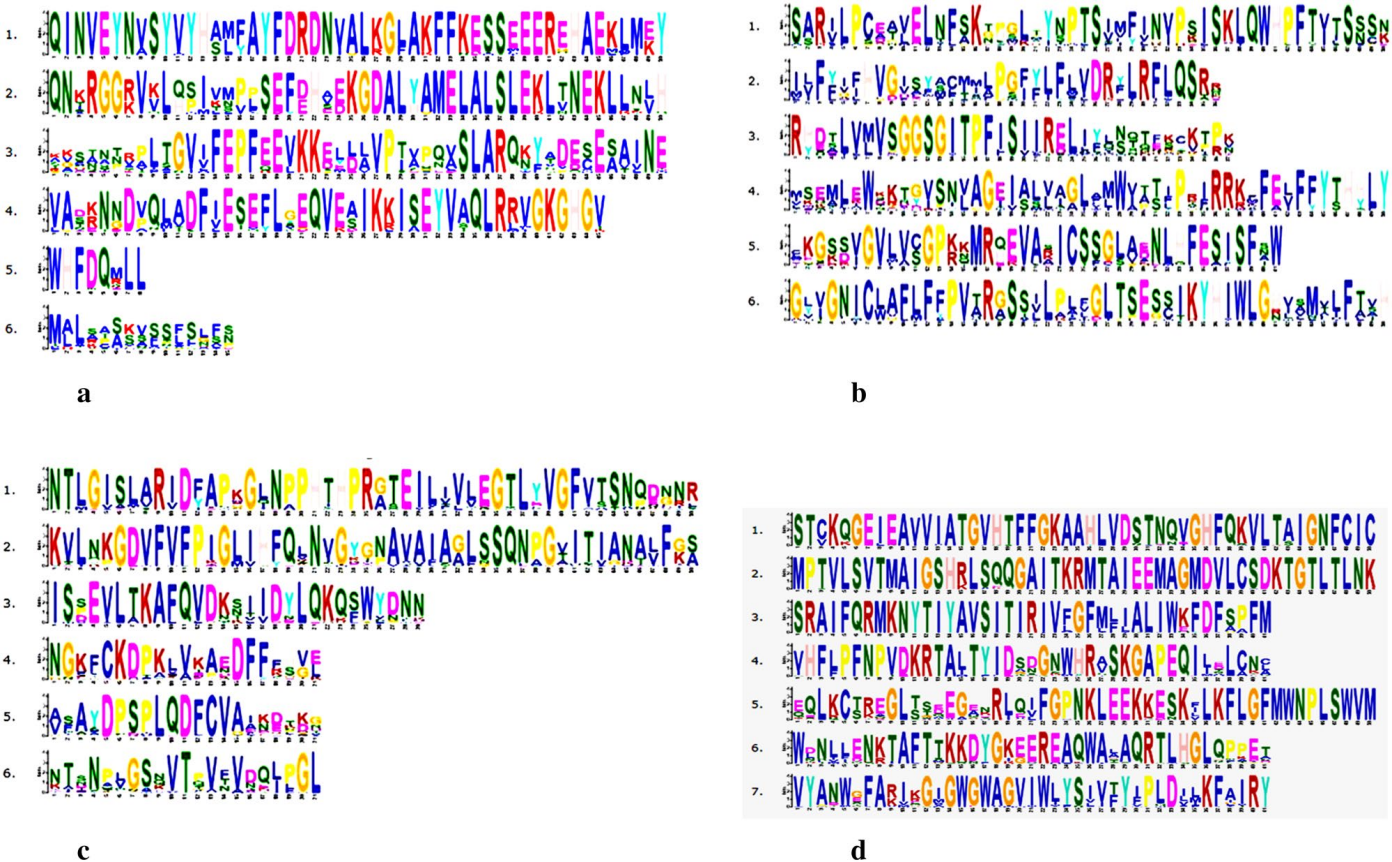
Sequence logo of the six most conserved motifs in FER (**a**), FRO (**b**), GLP (**c**) and HA2 (**d**) in *Phaseolus vulgaris* L*.* and other dicot plant species .

**Figure 3:**
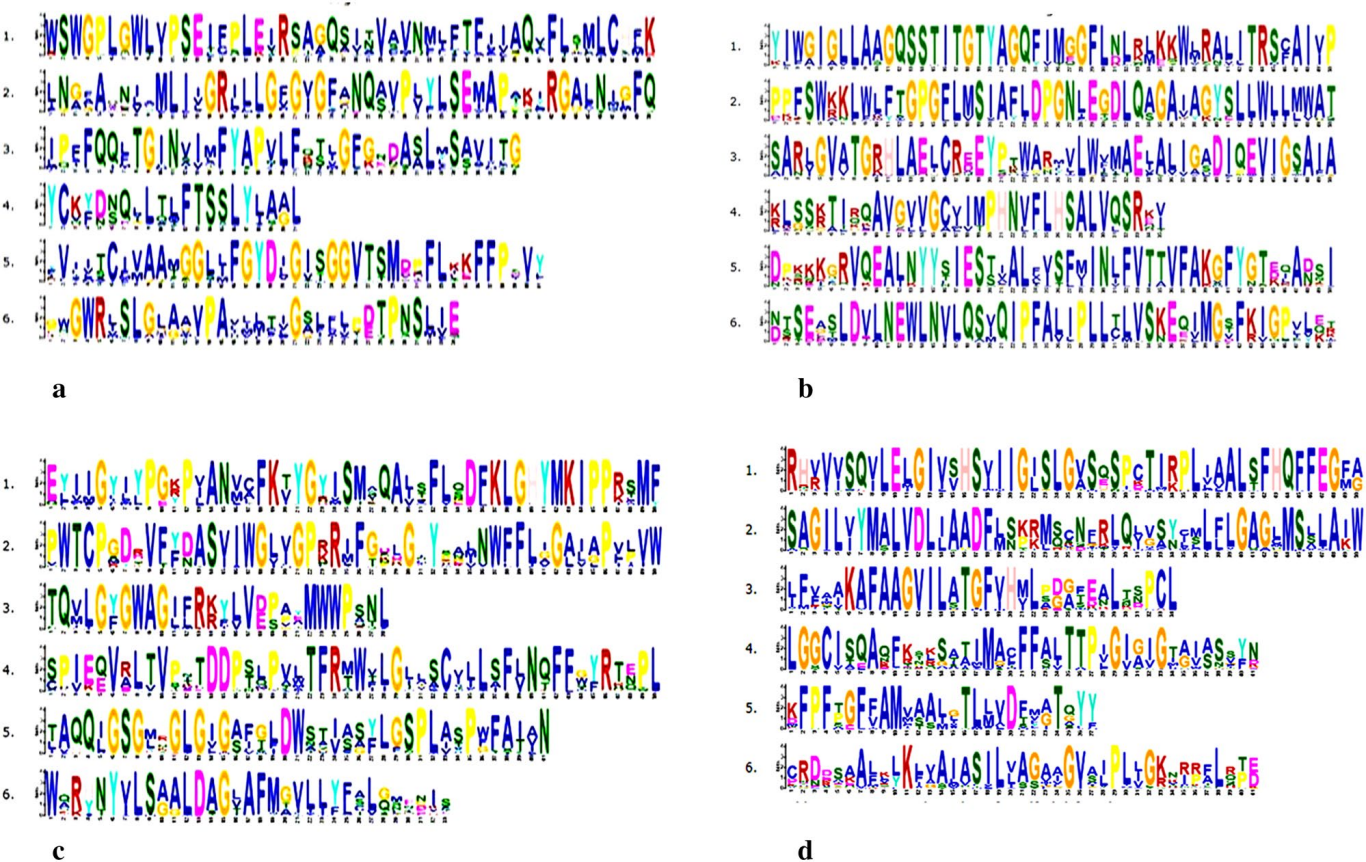
Sequence logo of the six most conserved motifs in IRT (**a**), NRAMP (**b**), OPT (**c**) and ZIP (**d**) in *Phaseolus vulgaris* L*.* and other dicot plant species .

Motif analysis was performed by using the MEME motif search tool to identify most conserved six motif types in 10 Fe/Zn stress responsive proteins of *Phaseolus vulgaris* and other dicot plant species. These motifs for each proteins are present in almost all respective sequences proposing that motif structures for Fe/Zn responsive proteins are well conserved in dicot plant species and is given for: FER (Fig. 2a), FRO (Fig. 2b), GLP (Fig. 2c) HA2 (Fig. 2d) IRT (Fig. 3a), NRAMP (Fig. 3b), OPT (Fig. 3c) and ZIP (Fig. 3d).

### Chromosomal localisation of Fe/Zn stress responsive genes

**Figure 4:**
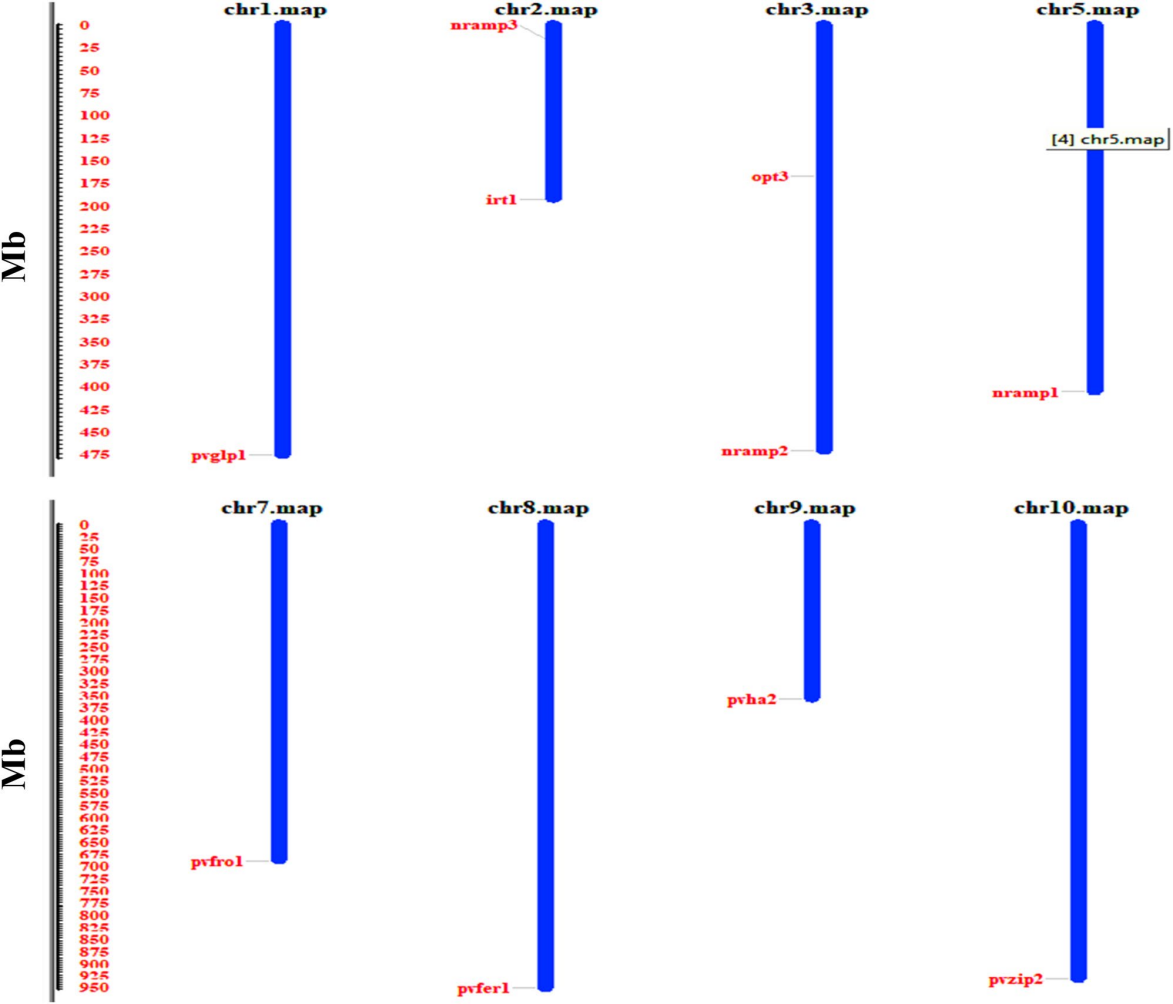
Chromosomal localization and distribution of Fe/Zn responsive genes in *Phaseolus vulgaris* L. The graphical view was drawn from each gene ID and scaffolds information and position of each gene is indicated by line, whereas scale bar represents the total length of chromosome in megabases (Mb). The figure was generated by mapinspect software.

The chromosome location and annotation information of the Fe/Zn responsive genes in the common bean genome showed that Fe/Zn responsive genes are randomly distributed among the eleven chromosomes (Fig. 4) except chromosome 4, 6 and 11 Fe/Zn responsive genes were identified on all chromosomes.

### Gene structure analysis of Fe/Zn responsive gene

**Figure 5:**
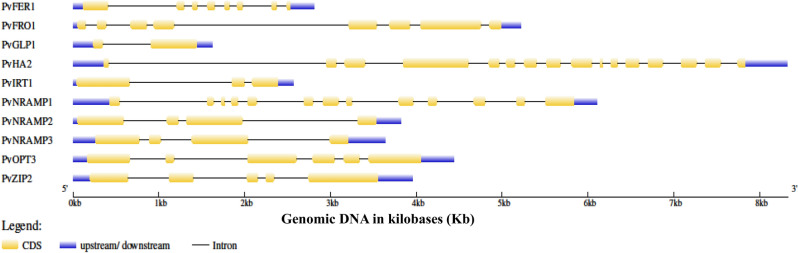
Gene architecture of Fe/Zn responsive genes in *Phaseolus vulgaris* L*.* The exon–intron structures of Fe/Zn responsive genes were determined by comparing the coding sequences and the corresponding genomic DNA sequences in kilobases (Kb) using the Gene Structure Display Server (GSDS). The yellow rounded rectangles indicate exons, the black lines indicate introns and the blue lines indicate upstream/downstream regions of gene.

To understand the structural characteristics of the Fe/Zn responsive genes, the exon–intron structures (Fig. 5) of Fe/Zn responsive genes were analyzed. Gene structure analysis revealed that the Fe/Zn responsive genes varied greatly in terms of gene structure. The number of exons and introns present in each Fe/Zn responsive genes are given in Table 1.

### Fe and Zn concentrations

Fe concentration in shoots of plants grown under 0-Zn was significantly higher (P ≤ 0.05) and was followed by Fe concentration in shoots of plants grown under 300-Zn, 0-Fe and control. A significant (P ≤ 0.05) difference in Fe content among shoots of plants grown under 4 different treatments was observed (Table 3 and Fig. 7a). Concentration of Fe in roots was significantly (P ≤ 0.05) higher in 0-Zn and lower in 0-Fe. The differences in Fe concentration of roots were significant (P ≤ 0.05) among treatments. The Fe concentration differences between shoot and root under different treatments were also significant (P ≤ 0.05). Concentration of Fe in shoots were significantly (P ≤ 0.05) higher in 0-Zn, 300-Zn and 0-Fe than roots of the respective treatments where as concentration of Fe in roots was significantly (P ≤ 0.05) higher in control compared to control in case of shoots.

**Table 3:** Micronutrient response of common bean to Fe and Zn stress grown under in vitro conditions (values are in Mean ± SE).

Concentration		Iron Conc. (ppb)		Zinc Conc. (ppb)	
S.No	Treatments	Shoot	Root	Shoot	Root
1	Control	126.362^a^ ± 0.654	188.012^a′^ ± 0.442	58.248^a^ ± 0.751	104.435^a′^ ± 1.525
2	0-Fe	185.584^b^ ± 0.701	79.646^b′^ ± 0.672	72.517^b^ ± 0.850	106.453^a′^ ± 1.199
3	0-Zn	318.378^c^ ± 0.778	210.630^c′^ ± 2.515	69.235^c^ ± 0.599	83.235^c′^ ± 0.997
4	300-Zn	224.120^d^ ± 0.932	170.641^d′^ ± 1.107	1137.748^d^ ± 1.171	7124.097^d′^ ± 2.151

The results are means of 12 biological replicates with three technical replicate for each treatment and are presented as Mean ± SE. Different superscript (a, b, c, d) denotes significant differences among treatments (P ≤ 0.05).

In shoots the average concentration of Zn was significantly (P ≤ 0.05) much higher in 300-Zn and lower in control. There was significant (P ≤ 0.05) difference among treatments (Table 3 and Fig. 7b). In roots concentration of Zn was significantly (P ≤ 0.05) higher in 300-Zn as compared to other treatments and lower in 0-Zn. The difference in concentration of Zn was non significant (P-value ≥ 0.05) between control and 0-Fe treatments and were significantly lower than other treatments whereas Zn concentration in 0-Zn and 300-Zn were significantly (P ≤ 0.05) different in comparison to other treatments. The difference in concentrations of Zn between shoot and root of different treatments were significant. The concentrations of Zn in roots of all the treatments were significantly (P ≤ 0.05) higher than the concentration of Zn of shoots.

### Differential expression of Fe /Zn responsive genes

The relative expression of the Fe/Zn responsive genes in terms of mean fold expression (2^−ΔΔCt^) of both tissues (shoot and root of *Phaseolus vulgaris* L.) under four different treatments is shown as in numerical data and graphical representation for fold expression of nine Fe/Zn responsive genes. The differences in fold expression levels of most of the genes in shoots are non significant (p ≥ 0.05) whereas in roots are highly significant (responsive).

### Iron regulated transporter 1 (IRT1) gene

**Figure 6:**
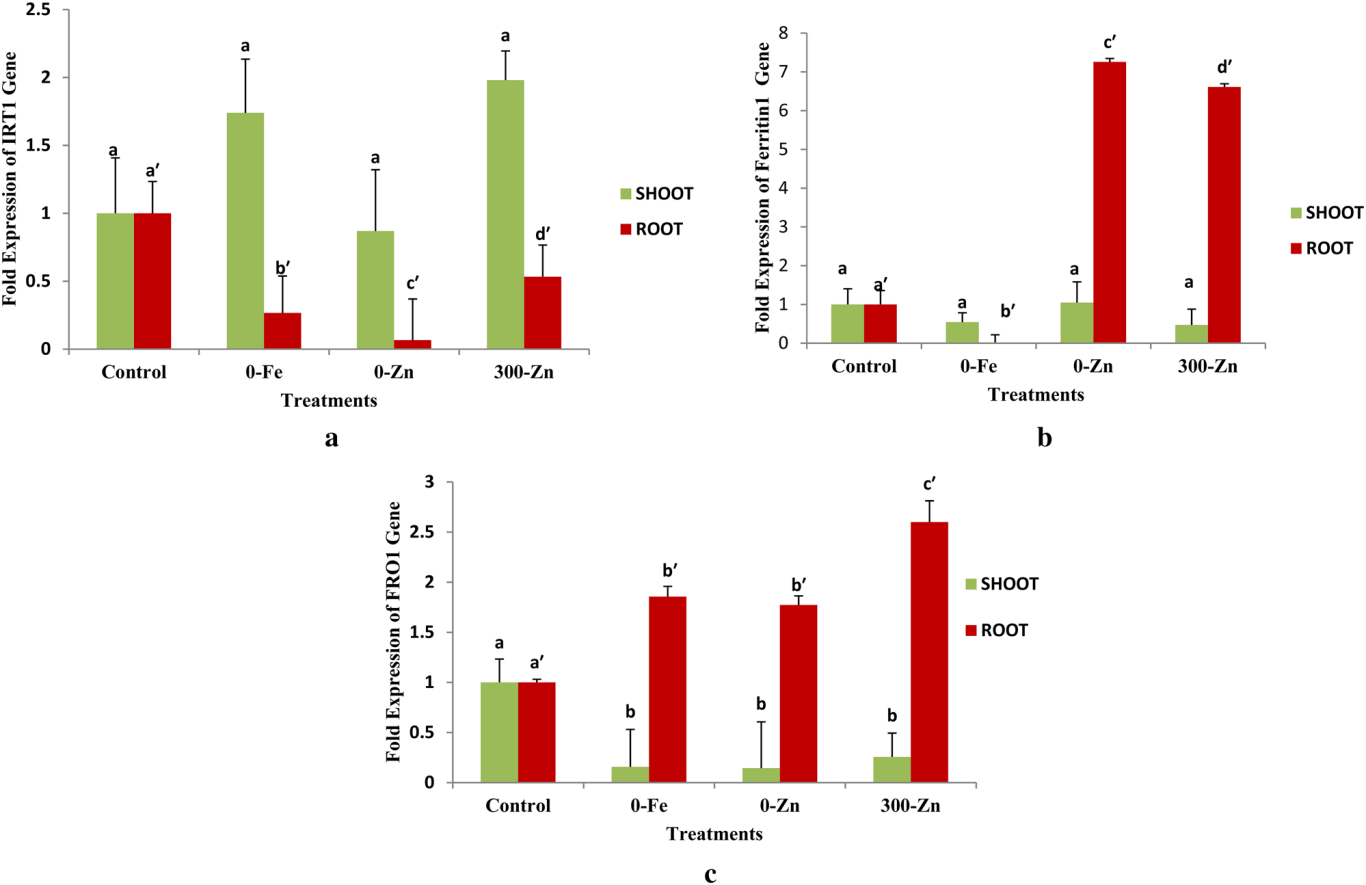
mRNA expression of IRT1 (**a**), Ferretin1 (**b**), and FRO1 (**c**) of shoot and root tissues due to different treatments in *Phaseolus vulgaris* L. under in vitro conditions.

Differential expression in terms of mean fold expression of IRT1 gene in shoots under Fe/Zn stress was non significant (p > 0.05) (Fig. 6a and Supplementary Table 2). Whereas mRNA expression levels of IRT1 gene in root was significantly (p < 0.05) down regulated in 0-Zn (0.066 fold), 0-Fe (0.266 fold) and 300-Zn (0.533 fold) with respect to control (1.000 fold) and was also significantly different among all treatments.

### Ferritin1 (FER1) gene

mRNA expression levels of Ferritin1 gene (Fig. 6b and Supplementary Table 3) in shoots under Fe/Zn stress was found non significant (p > 0.05) but in roots mRNA expression levels of Ferritin gene was highest in control (1.000 fold) and least expression was found in 0-Fe (0.012 fold) followed by 0-Zn (0.090 fold) and 300-Zn (0.082 fold). Similar expression levels were found in roots of 0-Zn and 300-Zn.

### Ferric reduction oxidase 1 (FRO1) gene

mRNA expression levels of FRO1 gene in response to Fe/Zn stress (Fig. 6c and Supplementary Table 4) in shoot was significantly (p < 0.05) down regulated in 0-Fe (0.157 fold), 0-Zn (0.144 fold) and 300-Zn (0.256 fold) with respect to control (1.000 fold). In roots mRNA expression levels of FRO1 gene was significantly (p < 0.05) up regulated in 300-Zn (2.6 fold) followed by 0-Fe (1.855 fold) and 0-Zn (1.772 fold) as compared to control (onefold). The expression levels of FRO1 gene in 0-Fe and 0-Zn was found similar.

### Oligopeptide transporter 3 (OPT3) gene

**Figure 7:**
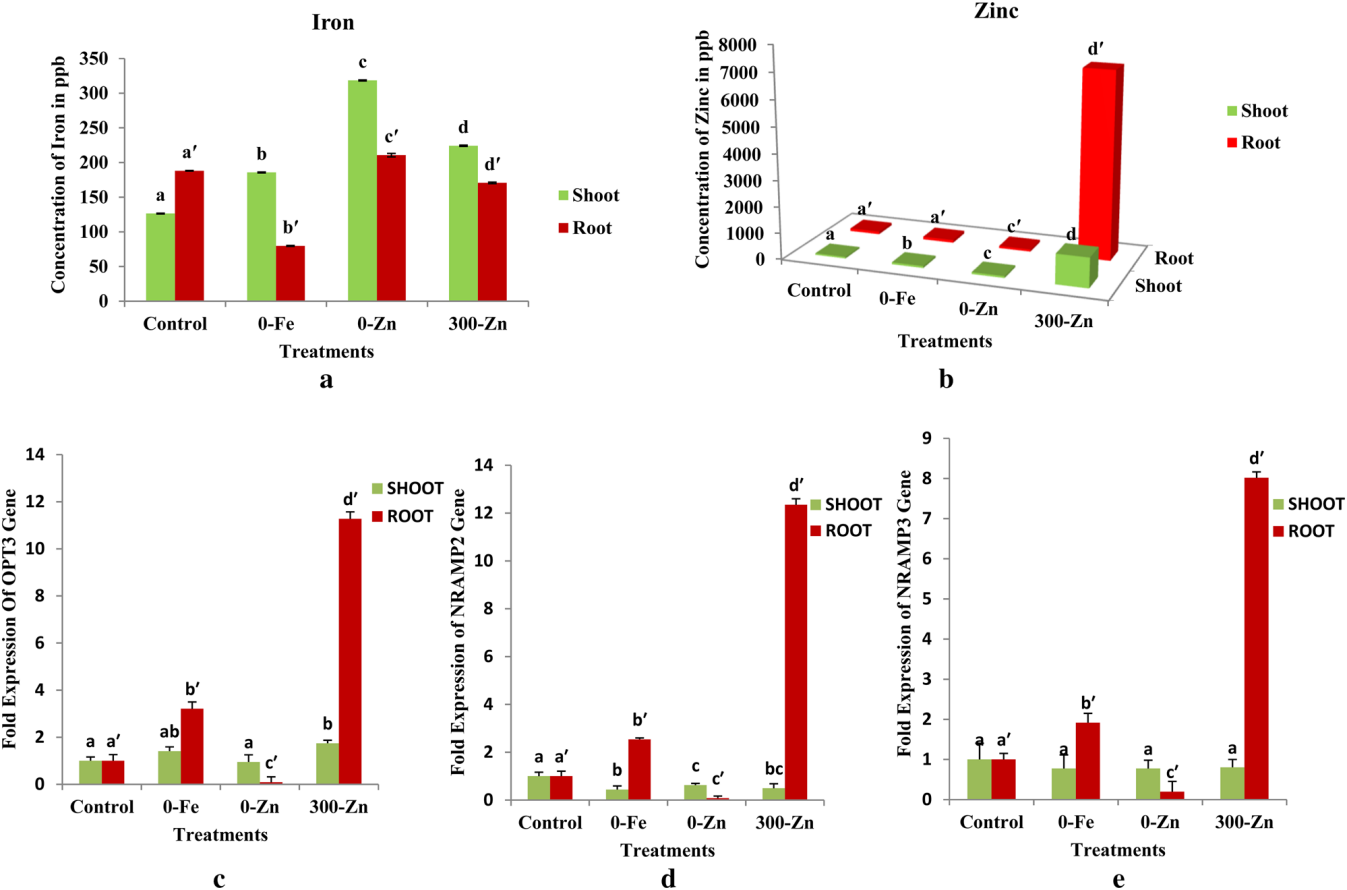
Micronutrient content (Iron) (**a**) and Zinc (**b**) and mRNA expression of OPT3 (**c**), NRAMP2 (**d**) and NRAMP3 (**e**) of shoot and root tissues due to different treatments showing cross talk between Fe-deficient and excess Zn conditions in *Phaseolus vulgaris* L. under in vitro conditions.

Expression of OPT3 gene (Fig. 7c and Supplementary Table 5) in shoot was significantly (p < 0.05) higher in 300-Zn (1.742 fold) then control (1.000 fold), 0-Fe (1.408 fold) followed by 0-Zn (0.948 fold). Whereas in roots mRNA expression levels of OPT3 gene was found significantly (p < 0.05) up regulated in 300-Zn (11.272 fold) and 0-Fe (3.208 fold) but huge dip in fold expression was observed in 0-Zn (0.087 fold) with respect to control (1.000).

### Natural resistance associated macrophage protein 1 (NRAMP1) gene

**Figure 8:**
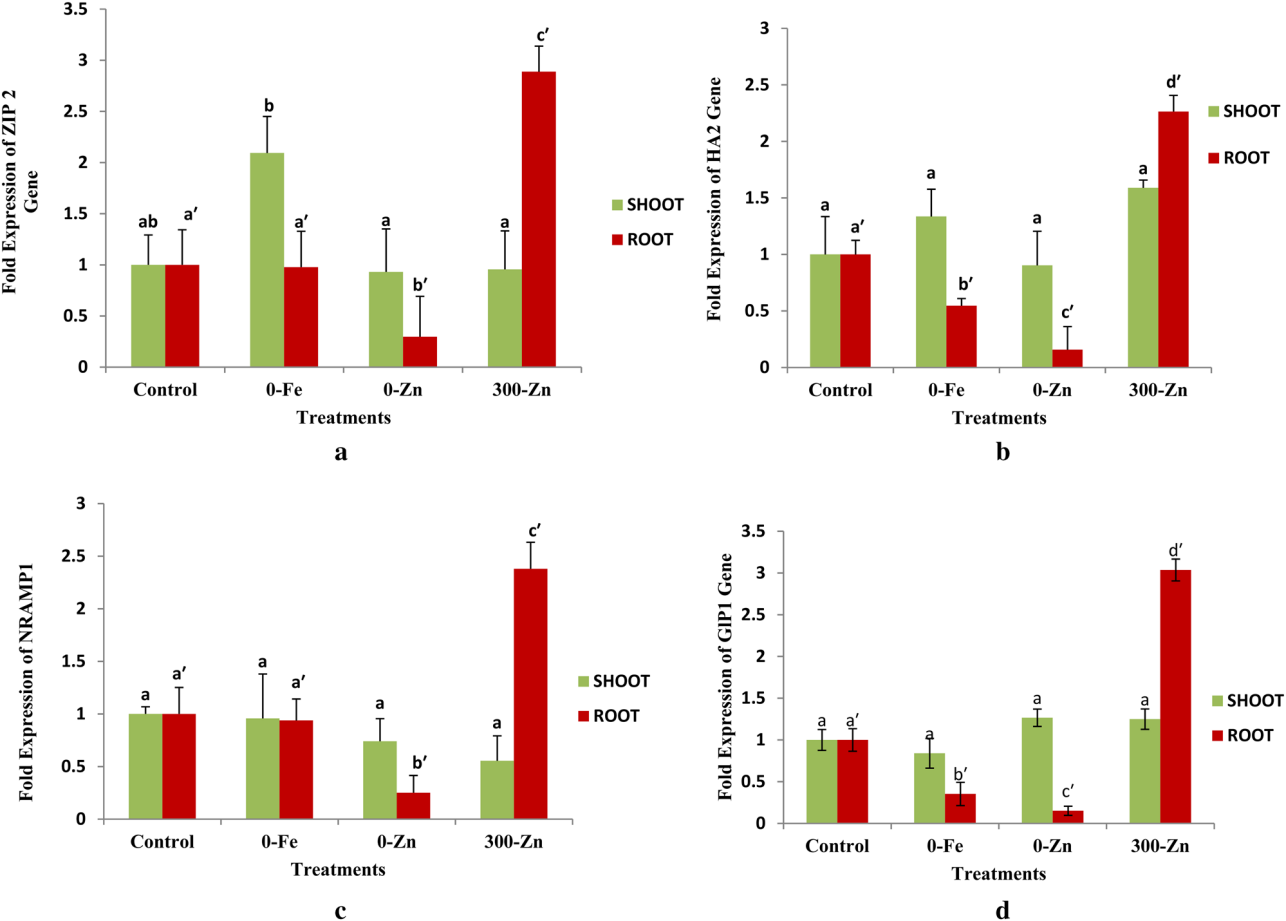
mRNA expression of ZIP2 (**a**), HA2 (**b**), NRAMP 1 (**c**) and GLP1 (**d**) of shoot and root tissues due to different treatments confirming their role in Zn uptake /transport in *Phaseolus vulgaris* L. under in vitro conditions.

Expression of NRAMP1 gene (Fig. 8c and Supplementary Table 6) under Fe/Zn stress was non significant (p > 0.05) in shoot whereas in roots it was found to be significantly (p < 0.05) upregulated in 300-Zn (2.383 fold) and down regulated in 0-Zn (0.251 fold) as compared to control (1.00 fold). The difference in expression levels of NRAMP1 gene between control (1.000 fold) and 0-Fe (0.938 fold) was found to be non significant (p > 0.05) in roots.

### Natural resistance associated macrophage protein 2 (NRAMP2) gene

NRAMP2 gene expression level (Fig. 7d and Supplementary Table 7) in response to Fe/Zn stress was significantly (p < 0.05) lower in 0-Fe (0.438 fold), 300-Zn (0.493 fold) and 0-Zn (0.624 fold) followed by control (1.000 fold) in case of shoots. mRNA expression levels of NRAMP2 gene in roots was found to be significantly (p < 0.05) up regulated in 300-Zn (12.346 fold) and 0-Fe (2.536 fold) and a huge dip in fold expression was found in 0-Zn (0.080 fold) with respect to control (12.432 fold).

### Natural resistance associated macrophage protein 3 (NRAMP 3) gene

NRAMP3 gene expression levels (Fig. 7e and Supplementary Table 8) under Fe/Zn stress was non significant (p > 0.05) in shoots whereas in roots mRNA expression levels of NRAMP3 gene under stress conditions was found significantly (p < 0.05) up regulated in 300-Zn (8.017 fold) and 0-Fe (1.916 fold) but down regulated in 0-Zn (0.195 fold) with respect to control (1.000 fold).

### Zinc induced protein 2 (ZIP2) gene

Expression levels of ZIP gene (Fig. 8a and Supplementary Table 9) in shoots was significantly (p < 0.05) up regulated in 0-Fe (2.093 fold) compared to control (1.000), 0-Zn (0.931 fold) and 300-Zn (0.956 fold). mRNA expression levels of ZIP gene in root was significantly (p < 0.05) up-regulated in 300-Zn (2.888 fold) and down regulated in 0-Zn (0.297 fold) but non significant (p > 0.05) difference was found between control (1.000 fold) and 0-Fe (0.977 fold).

### H^+^ transporting ATPase2 (HA2) gene

Expression levels of HA2 gene (Fig. 8b and Supplementary Table 10) in response to Fe/Zn stress was found to be non significant (p > 0.05) in shoots whereas in roots it was significantly (p < 0.05) up regulated in 300-Zn (2.264 fold) but down regulated in 0-Fe (0.546 fold) and 0-Zn (0.157) with respect to control (1.000 fold) and levels of expression was significantly (p < 0.05) different among treatments.

### Germin like protein 1 (GLP1) gene

Expression of GLP1 gene (Fig. 8d and Supplementary Table 11) was found to be non significant (p > 0.05) in shoots under Fe/Zn stress response in *Phaseolus vulgaris* L*.* whereas expression levels in roots was significantly (p < 0.05) up regulated in 300-Zn (3.036 fold) and down regulated in 0-Fe (0.353 fold) and 0-Zn (0.151 fold) in comparison to control (1.000 fold).

### Protein–Protein Networks Analysis of the Fe/Zn responsive Genes

**Figure 9:**
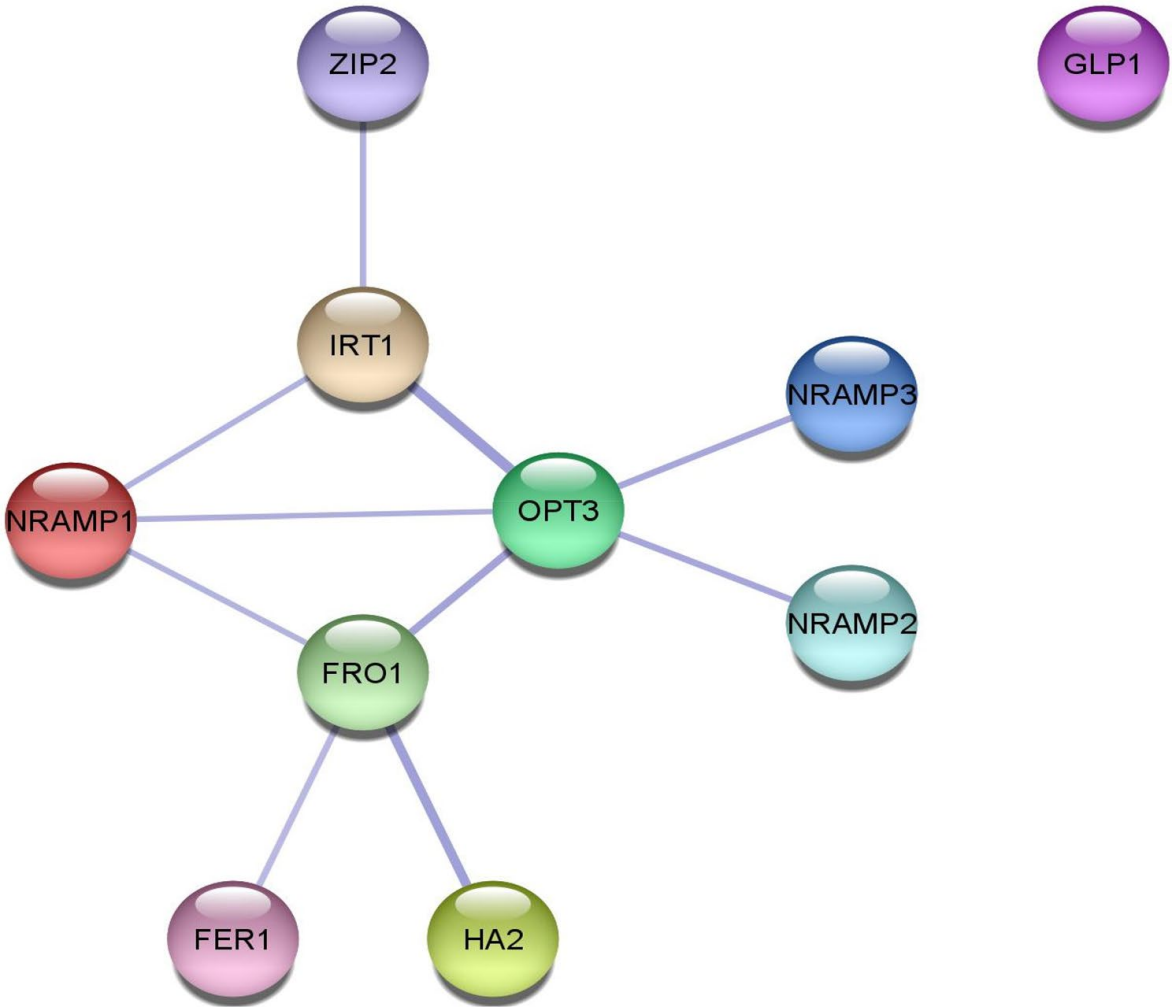
Protein–protein interaction analysis of Fe/Zn responsive proteins. Protein–protein interaction network produced by STRINGV9.1. Each EDGE represents an Interaction.

Using the STRING database (Fig. 9), we here framed protein–protein interactions among 10 Fe/Zn responsive proteins, accordingly we observed that except GLP1, other nine Fe/Zn responsive proteins interact with one another. OPT3 was observed interacting with 5 Fe/Zn responsive proteins such as NRAMP1, NRAMP2, NRAMP3 IRT1and FRO1, while FRO1 was found interacting with FER1, HA2, OPT3 and NRAMP1. IRT1 was shown interacting with ZIP2. The interaction among these Fe/Zn responsive proteins indicate their possible function in the uptake and homeostasis of Fe and Zn in *Phaseolus vulgaris* L.

## Discussion

### In silico identification of Fe and Zn responsive genes

Identification of Fe/Zn stress responsive genes has a great significance in improving accumulation of these nutrients in crop plants. Identification and characterization of Fe/Zn responsive genes in common bean through genetic screening and direct cloning is difficult due to the non-availability of specific genomic loci information data and the tissue/time specific expressions of some genes^21^. Computational approach is a powerful tool that has simplified the identification and characterization of potential candidate genes for abiotic stresses^21^. The current study was performed for identification of mineral (Fe/Zn) stress responsive genes in *P. vulgaris* through in silico tools. Similar studies have been carried out in a wide variety of plant species and a large number of stress responsive genes have been identified in these species^22^. Genes responding under various abiotic stresses like; cold, heat, drought and salt stresses were identified as cumulative abiotic stress responding genes by using BLASTx and BLASTn tools in potato^22^. The in silico research using bioinformatics tools are a rational approach to find interesting findings^23–25^. In silico searches of many plant genomes or proteomes was conducted to find abiotic stress responsive genes like NRAMP genes in *Glycine max*^26^, glutathione S-transferase genes in *Vigna radiata* L.^27^, proteases in *Cicer arietinum*^28^ and so on.

In the present study, we unraveled most of the topological features and physicochemical properties of identified Fe/Zn stress responsive transporter/gene sequences that were showing consistency with the current literature. Overall, we observed that information about gene and protein features of Fe/Zn stress transporter proteins could be used in identification of Fe/Zn stress responsive transporter homologs in different plant genomes. The trans-membrane helices and subcellular localization was also predicted by using various online bioinformatic tools. Some of identified Fe/Zn responsive proteins are localized to cell membrane, mitochondria, chloroplast and vacuole providing support for their function as metal transporter or tolerance against stresses. Those localized to nucleus function as transcription factors^29^. Such compartmentalization is likely related to specific functions, namely, uptake of metals on the plasma membrane, or release from tonoplast as previously reported in Arabidopsis and rice^26^. Protein motifs for Fe/Zn stress responsive protein were generated by using MEME suite. Motif analysis was performed for the identification of most conserved six motif types for each Fe/Zn stress responsive protein. MEME analysis suggested that motifs found were present in most of the sequences of each protein taken into consideration. This analysis suggests that motif structures of Fe/Zn stress responsive proteins are well conserved in dicot plants and may be used as a base for protein classification.

The multigene phylogenetic tree generated by MEGA 7.0v was grouped into two clusters- dicots and monocots and all the dicot plants used in the study got clustered into one group. This suggested differentiation among these proteins following the divergence of monocots and dicots from a common ancestor. This phylogenetic analysis of Fe/Zn responsive genes among the 20 plants showed that these proteins have high level of conservation in dicot plants. These results suggested that phylogenetic analysis implied that Arabidopsis (model dicot crop) or any other dicot plant, Fe/Zn responsive transporter/gene sequences could be used as references/benchmarks to identify the corresponding homologs of Fe/Zn transporters in various other dicot plant species. This analysis also showed *Glycine max* and *Phaseolus vulgaris* are closely related as depicted in results.

Using a combined computational strategy, the current study identified 10 Fe/Zn responsive stress genes in the *Phaseolus vulgaris* genome and characterized them based on their subcellular localization, gene structure, motif analysis, and phylogeny. The results of the current study can potentially be used for functional characterization of Fe/Zn responsive stress and detailed investigation of the Fe/Zn responsive stress gene family. The role of Fe/Zn responsive stress tolerance genes can be elucidated in further studies and the candidate genes identified can be used for development of mineral stress tolerant crop plants with enhanced productivity.

### Fe and Zn partitioning during early growth stage

Different response to micronutrient shortage was observed in both tissues of common bean grown under in vitro conditions. We observed higher accumulation of Fe in shoots under 0-Fe condition compared to control. However, the total concentration of Fe in plant under 0-Fe was less compared to the concentration of Fe in plants grown under control condition. Similarly concentration of Zn was less in roots and more in shoots of 0-Zn compared to control but the total concentration of Zn in plants under 0-Zn was less compared to plants grown under control conditions. The differences in concentration of these micronutrients between shoot and root suggests precise sensing for priority based compartmentalization and partitioning leading to higher accumulation of these micronutrients in shoots under mineral stress. These are the mechanisms developed by the plants under mineral stress both at the cellular and systemic levels called plant metal homeostasis to balance the concentrations of micronutrients that are involved in central cellular processes of plants including compartmentalization and partitioning, daily redox oscillations, or transcriptional regulation of micronutrients. The intracellular compartmentalization and partitioning of metals seems essential for optimizing the use of micronutrients during development and in response to deficiencies^4^. We also found higher accumulation of Fe and Zn in shoot and root of plants under 0-Zn and 0-Fe respectively. As Fe and Zn, are divalent elements, they show antagonistic behavior during absorption. Therefore, a change in concentration of any one element in a nutrient solution could affect uptake of other elements. Generally some transporters are involved in uptake these elements^30^. The reasons for antagonistic effects of Fe and Zn are competition for uptake by transporters located on root cells to enter into xylem cells and disorder in metal chelating process in roots^31^. Concentration of Zn was found excess in both shoot and root of 300-Zn because of more Zn present in 300-Zn treatment compared to other treatments resulted in hyper accumulation of Zn in both the tissues of plants under 300-Zn treatment and leads to Zn toxicity in plants.

### Differential expression of IRT1, FRO1 and Ferritin 1 suggests their role in metal homeostasis

Three candidate genes among the nine selected genes have shown same pattern of expression in all 3 treatments; (0-Fe, 0-Zn and 300-Zn) for both the tissues. IRT1 and Ferritin gene was down regulated in roots of all 3 treatments whereas FRO1 was up regulated in roots and down regulated in shoots of all 3 treatments. Iron regulated transporter 1 (IRT 1) is a major player in the regulation of plant Fe homeostasis and is completely down regulated when Fe is completely lacking^32^. Fe deficiency in Arabidopsis up regulates the expression of IRT1, the primary transporter responsible for root Fe uptake. IRT1 also contributes to the accumulation of a broad range of divalent transition metals including Zn because of its weak substrate specificity^33–35^. Conversely, excess Zn causes physiological Fe deficiency. Early studies reported an absence of IRT1 protein in Arabidopsis roots from plants grown under Zn excess conditions^36,37^, suggesting ubiquitin-mediated proteasomal degradation of IRT1 protein which is the known post-translational regulation^38,39^. This is in consistent with the observation that the expression levels of the IRT1 gene in roots under Fe and Zn stress conditions were down regulated in the present study.

FRO1 gene was up regulated to 1.855-, 1.772- and 2.600- fold in response to 0-Fe, 0-Zn and 300-Zn respectively in roots of *Phaseolus vulgaris* but was down regulated in shoots of all stressed plants that suggests its role in metal homeostasis*.* Ferric reductase oxidase (FRO2 in *A. thaliana*; LeFRO1 in tomato) is responsible for the reduction of Fe^3+^ to Fe^2+^ which is a crucial step for the Fe uptake in Strategy I plants^40–42^. LeFRO1 mRNA is also detected in shoots regardless of Fe status, suggesting that LeFRO1 may play a role in Fe mobilization and responsive to Fe status in the shoots as well. In onion epidermal cells, LeFRO1 localizes to the plasma membrane and confers Fe (III) reductase activity when expressed in yeast^43^. In root tissues, the expression of FRO1 mRNA is consistent with its proposed role in reduction of rhizosphere Fe (III). Expression of FRO1 gene was low in Fe-sufficient shoots, but was elevated in shoots of Fe-deficient plants. These results suggest a role for Fe (III) reduction in leaf tissues^44^. In the present study Fe is sufficient in shoots of stressed plants which was also confirmed by elemental analysis of shoots which supports down regulation of FRO1 in shoots compared to control conditions. In some previous experiments, FRO1 mRNA in the vascular bundles of leaves was not highly expressed, indicating that other proteins or transport mechanisms may be at work mobilizing xylem Fe (III). A Fe (III)-chelate reductase activity of FRO1 and its expression in chloroplast-containing cells has suggested to aid uptake of intracellular Fe by chloroplasts^45^. These results indicated that expression of FRO1 in shoots and roots is affected by different signals or Fe-sensing mechanisms. FRO1 regulation in shoots appears to respond to the Fe status of these tissues.

Ferritin1 (FER1) gene expression in roots was decreased to 0.012-, 0.090- and 0.082- fold for 0-Fe, 0-Zn and 300-Zn condition respectively compared to normal condition. Ferritins (FER), functioning as ferric iron binding and participating in the cellular Fe homeostasis, are essential to protect cells against oxidative damage and flowering^46^ and is modulated by many abiotic and biotic stresses (nutrient deficiency, stress, and microorganism attack) in plants^47,48^. FER1 was identified to be robustly down-regulated in roots of Arabidopsis under Fe deficiency^49–51^ and this finding is in agreement with the present study. mRNA expression of FER gene was seen in the vascular cylinder of the mature root hair zone^43^. The down regulation of FER1 gene in stressed conditions could be attributed to that Fe/Zn stress altered nutrient status of the stressed plants that leads to modulation in gene expression of FER1.These results suggests that common regulation of these three genes under Fe/Zn stress have role in uptake/transport and signaling network of Fe and Zn status in *Phaseolus vulgaris*.

### Differential expression of OPT3, NRAMP2 and NRAMP3 reveals possible cross talk between Fe-deficient and excess Zn

Among nine genes, we observed that the expression of three identified candidate genes (OPT3, NRAMP2 and NRAMP3) was higher in 0-Fe and 300-Zn media. The common regulation of these three genes in response to Fe deficiency and excess Zn induced iron deficiency indicates regulatory cross talk among these two conditions. Oligopeptide transporter 3 (OPT3) was upregulated 3.208 and 11.272 fold in roots of plants grown on 0-Fe and 300-Zn media respectively. OPT3 is involved in the transport of small peptides that may have roles in nutrition^52^. OPT3 plays a critical role in the maintenance of whole-plant Fe homeostasis and Fe nutrition in developing seeds and it plays an important role in shoot to root signaling for the regulation of Fe deficiency responses in roots^53^. Stacey et al.^54^ showed that OPT3 expression was enhanced by Fe limitation and excess Zn. The high level of OPT3 expression under Fe-deficient and excess-Zn conditions shown in this study provides further support for the role of this gene in defense against Fe deficiency stress and partitioning of Fe content between root and shoot in *Phaseolus vulgaris*. OPT3 mediates partitioning of Fe from source to sink tissues^55^. Therefore, to determine the contribution of OPT3 to Fe partitioning, we examined concentrations of Fe in shoot (sources) and root (sinks) of the Fe/Zn stressed plants. We found higher concentration of Fe content in shoots than roots of 0-Fe and 300-Zn. Our findings suggest that OPT3 may have role in both signaling of Fe demand from shoots to roots and Fe transport to developing tissues. This data also showed an aspect of crosstalk between Fe homeostasis and partitioning that is mediated by OPT3.

Natural resistance associated macrophage protein 2 (NRAMP 2)/DCT1 (divalent cation transporter 1)/DMT1 (divalent metal transporter 1) was also highly expressed under both 0-Fe and 300-Zn growth conditions. NRAMP 2 was up regulated to 2.536- and 12.346—fold in roots of 0-Fe and 300-Zn, respectively. NRAMP2 has a role in transport of Fe/Zn and may have a role in tolerance to mineral stress. Gao et al.(2018)^56^ found that NRAMP2 has role in transport of Mn, Fe and Zn in yeast and is involved in remobilization of Fe in roots. NRAMP 3 (natural resistance associated macrophage protein 3) gene expression was also increased to 1.916- and 8.017- fold in case of 0-Fe and 300-Zn respectively. AtNRAMP3, a multi-specific vacuolar metal transporter protein are up regulated at the transcriptional level by Fe deficiency and mediates the release of Fe from the vacuole and provide sufficient Fe during seed germination^57–59^. NRAMP genes are up-regulated under Fe deficient conditions to regulate Fe nutrition. It was found that, under stress conditions, NRAMP acts as mineral regulatory element and defends plants against stresses^60^. These findings suggest its role in crosstalk and assisting the transport of Fe/Zn under Fe-deficient and Zn-excess conditions. These genes may serve as backup systems for Fe homeostasis. However, further evidence is needed to confirm the mechanism underlying increased NRAMP genes expression under Fe-deficient conditions.

### Zn transport responsive genes

Three candidate genes/transporters-ZIP2, NRAMP1, HA2 and GLP1 were found up and down regulated in roots of stressed plants under 300-Zn and 0-Zn conditions respectively. ZIP2 gene was up-regulated to 2.888-fold and down-regulated to 0.297-fold under excess-Zn and Zn deficient conditions respectively that indicates its role in Zn uptake in roots. Root and shoot *AtZIP2* transcript abundance was decreased in response to Zn, Fe, and Mn deficiency in Arabidopsis^61^. ZIP genes revealed their role in regulating the uptake, transport and accumulation of Zn in Arabidopsis^61–65^. In Arabidopsis at least 10 different members of the ZIP family play a role in Zn uptake in roots, including ZIP1, 2, 3, 4, 5, 9, 10, 11, 12, and IRT3^66^. The expression of natural resistance-associated macrophage protein 1 (NRAMP1) gene, was increased to 2.383-and decreased to 0.251-fold under 300-Zn and 0-Zn conditions respectively. Members of the NRAMP family are functional divalent metal ion transporters^67^ that are conserved in different species and located in the plasma membrane of root apical cells^68^. They are involved in proton-coupled active transport of various heavy metals (Fe^2+^, Zn^2+^, Mn^2+^, Co^2+^, Cd^2+^, Cu^2+^, Ni^2+^, and Pb^2+^) in wide range of organisms including bacteria, fungi, animals, and plants^69,70^ but limited literature is available on how NRAMP proteins function to transport Zn in plants. Perhaps, AhNRAMP1 might be a functional transporter of other divalent metal ions such as Mn and Zn^71^. Wang et al.^71^ proposed AhNRAMP1 as a Mn and Zn transporter and an important candidate transporter for Fe and Zn biofortification. They reported, even under the excessive Zn supply in hydroponics, expression of AhNRAMP1 from peanut plants promote Zn concentration in transgenic plants and resulted in the risk of heavy metal accumulation. AtNRAMP1 and 6, forms the first group and AtNRAMP2–5 constitute the second group^72^. Of these, *At*NRAMP1, *3*, *4*, and *6* have been shown to encode functional plant heavy metal transporters^69,73^. These studies are in agreement with the results of the present study that NRAMP1 is involved in transportation/uptake of Zn.

H^+^-ATPase 2 pump (HA2) gene expression was increased to 2.264- and decreased to 0.157-fold in response to 300-Zn and 0-Zn respectively. HA2 is responsible for the major acidification activity. It is up-regulated in Fe deficiency^32^ and Zn induced Fe deficiency and is also increased in conditions requiring greater transport activity as more and more Zn is taken up under excess-Zn conditions in the present study which is confirmed by up-regulation of other Zn transporters like ZIP2 and NRAMP1. The external signal caused due to heavy metal stress results in change in HA2 gene expression to control the major transport processes in the plants such as nutrient uptake and xylem or phloem loading to play role in adaptation of plants to changing conditions of stress^74^. Excess-Zn may trigger a signal transduction pathway that up-regulates the expression of transporters to increase the efficiency of Zn transport and results in accumulation of Zn in plants under excess-Zn conditions. However, further evidence is needed to confirm the mechanism underlying increased expression of these genes under excess-Zn conditions.

In our study, we observed higher expression of Germin like protein 1 (GLP-1) gene in roots under excess Zn that reveals its role to overcome heavy metal stress. GLPs have been proposed to play a role in reactive oxygen species detoxification and to function as signaling molecules inducing a range of defense responses in a direct or indirect manner^75,76^. Germin like proteins (GLPs) include the first crystal structure of extracellular superoxide dismutase (SOD) which are implicated in the response of plants to many abiotic stresses such as exposure to heat, salt, submergence, and aluminum toxicity^77,78^, which provide evidence that GLPs represent a new family of extracellular SODs, leading to the generation of H_2_O_2_ in response to abiotic stress in plants^78,79^. In the present study higher accumulation of Zn in plants under 300-Zn (excess zinc) leads to oxidative stress in the roots of *Phaseolus vulgaris* and induces expression of GLP1 to overcome stress. GLPs members have been confirmed to have oxalate oxidase or superoxide dismutase functions which catalyze the oxalate degradation resulting in the production of hydrogen peroxide^80^, and Zn toxicity is a source of superoxide radicals that impose oxidative stress to plant cells. Li et al.^80^ studied expression of soybean germin-like gene family revealed a role of GLP7 gene in various abiotic stress tolerances. Peanut germin-like proteins (AhGLPs) plays role in plant development and defense confirmed by Wang et al.^81^ and also reported the genetic connection between GLPs and ABA-mediated stress responses. We presume that pvGLP1 is a good candidate gene for increasing tolerance to heavy metal stress in plants (a new finding) but further confirmation is required.

### Protein–protein networks analysis of the Fe/Zn responsive Genes

To regulate nutrient uptake and in response to different biotic and abiotic stress, it has been observed that Fe/Zn responsive genes interact with other proteins. In the present study, STRING database predicted results that revealed OPT3 interacts with NRAMP1, NRAMP2, NRAMP3, IRT1 and FRO1; while FRO1 interacts with FER1, HA2, OPT3 and NRAMP1. Further, IRT1 interacts with ZIP2. The interactions among Fe/Zn responsive proteins indicate their possible function in the uptake and regulation of Fe and Zn in *Phaseolus vulgaris*. Moreover, the interactions among these proteins supports that common bean deploy strategy I for the uptake of Fe and Zn. Strategy I is a reduction strategy, used by dicots and non-grass monocots, which involves proton pumps HA2 to acidify the rhizosphere surrounding the root, increasing the solubility of Fe (III), by involving ferric reduction oxidase (FRO). Iron regulated transporter 1 (IRT1)/(NRAMP1)/(DMT1/NRAMP2) is then involved in transport of Fe (II) through the assembly of metal transporters in plasma membrane of the epidermis. The results revealed interaction of IRT1 with ZIP2 for Zn transportation. In *Arabidopsis*, half of the zinc-regulated transporter, iron-regulated transporter proteins (ZIPs) family is induced under Zn deficient conditions^62,82^. As per the current model, based mostly on yeast studies^83^, the ZIP member IRT1 non selectively takes up Zn (as well as cadmium). This network of Fe/Zn responsive proteins showed interaction of OPT3 (An additional protein localized in the phloem) with IRT1, FRO1, NRAMP1, NRAMP2 and NRAMP3 as oligopeptide transporter 3 (OPT3), has been identified as a component of the shoot-to-root signaling network relaying the information of the Fe status in leaves to roots and might function in diverse stress tolerance in plants. Interestingly, since Fe and Zn share the same transporters to enter the root, mutants with impaired systemic signaling (*opt3* mutants) over accumulate Fe and Zn in roots and leaves ^55,84^. Mineral stress condition in plants leads them to evolve complex and well controlled network for uptake, distribution, and storage of minerals. This networks is the directed and controlled by many different genes including various mineral transporters, reductive agents, specialized storage proteins, metal ligands with different substrate specificities, and regulatory proteins such as transcription factors, protein kinase, and receptors^85^.

## Conclusion

The expression analysis of nine candidate genes revealed that there is more impact of mineral stress on root tissues. However, there is need to analyse more genes responsive to Fe/Zn stress to have holistic picture about regulation and stress tolerance. Further, the differential expression at proteome levels needs to be investigated to have a clear understanding about mineral stress tolerance and transport in common bean. This study demonstrates possible role of these candidate genes in mineral homeostasis and tolerance, suggesting their potential for use as a transgenes to induce mineral stress tolerance. However, functional characterization of these genes can also unravel their role in mineral fortification.

## Supplementary Information

Supplementary Information.

## Abbreviations

Fe: Iron
Zn: Zinc
ICP-MS: Inductively coupled plasma mass spectrometry
IRT: Iron regulated transporter
FER: Ferritin
FRO: Ferric reduction oxidase
OPT: Oligopeptide transporter
NRAMP: Natural resistance associated macrophage protein
ZIP: Zinc induced protein
HA: H^+^ transporting ATPase
