# Supplementary material for: Fe and Zn stress induced gene expression analysis unraveled mechanisms of mineral homeostasis in common bean (Phaseolus vulgaris L.)
Source: Sci Rep. 2021 Dec 15;11:24026. doi: 10.1038/s41598-021-03506-2 (PMC8674274; doi:10.1038/s41598-021-03506-2)
Supplement: Supplementary file 1 — Supplementary Information. [file 41598_2021_3506_MOESM1_ESM.docx]

**Supplementary Table 1: List of Fe/Zn responsive genes with their family/ superfamily profile IDs in different databases**

| **S.No** | **Genes identified** | **HMM Profile PFAM** | **PANTHER** | **InterPro** | **Domain** | **GO(Molecular)** | **GO(Biological)** | **Function** |
| --- | --- | --- | --- | --- | --- | --- | --- | --- |
| 1 | **FER** | PF00210 | PTHR11431 | IPR001519 | Ferritin like | GO:0008199 | GO:0006879 | cellular iron ion homeostasis,ferric iron binding |
| 2 | **FRO** | PF01794  PF08022  PF08030 | PTHR11972 | IPR013112  IPR013121  IPR013130  IPR017927  IPR017938 | FAD binding domain | GO:0016491 | GO:0055114 | oxidoreductase activity, oxidation-reduction process |
| 3 | **GLP** | PF07883 | PTHR31238 | IPR013096 | cupin | GO:0046564 | GO:0009409 | oxalate decarboxylase activity, response to abiotic stress |
| 4 | **HA2** | PF00122PF00690PF00702 | PTHR24093 | IPR004014 | - | GO:0046872 | GO:0006754 | metal ion binding,ATP biosynthetic process, role in proton extrusion under low iron availability. |
| 5 | **NRAMP1**  **NRAMP2**  **NRAMP3** | PF01566 | PTHR11706 | IPR001046 | - | GO:0006810 | GO:0071281 | metal ion transport, cellular response to iron ion |
| 6 | **OPT** | PF03169 | PTHR22601 | IPR004813 | tetrapeptide transporter | GO:0035673 | GO:0055085 | oligopeptide transmembrane transporter activity |
| 7 | **ZIP2/IRT1** | PF02535 | PTHR11040 | IPR003689 | - | GO:0005385 | GO:0071577 | zinc ion transmembrane transporter activity, zinc II ion transmembrane transport |

**Supplementary Table 2: Differential expression of IRT1 gene in response to Fe/Zn stress in *Phaseolus vulgaris* L*.***

| **Treatment** | **SHOOT** | | | | **ROOT** | | | |
| --- | --- | --- | --- | --- | --- | --- | --- | --- |
|  | **Mean Ct** | **ΔCt** | **ΔΔCt** | **2^-ΔΔCt^** | **Mean Ct** | **ΔCt** | **ΔΔCt** | **2^-ΔΔCt^** |
| **Control** | 25.410^a^±0.408 | 5.610^a^±0.408 | -0.202 | **1.000** | 17.360^a′^±0.234 | -4.640^a′^±0.234 | -3.906 | **1.000** |
| **0-Fe** | 24.617^a^±0.396 | 4.817^a^±0.396 | -0.995 | **1.739** | 19.230^b′^±0.272 | -2.770^b′^±0.272 | -2.036 | **0.266** |
| **0-Zn** | 25.612^a^±0.4529 | 5.812^a^±0.452 | 0.000 | **0.869** | 21.266^c^′±0.303 | -0.734^c′^±0.303 | 0.000 | **0.066** |
| **300-Zn** | 24.420^a^±0.215 | 4.620^a^±0.215 | -1.192 | **1.980** | 18.259^d′^±0.233 | -3.741^d′^±0.233 | -3.007 | **0.533** |

The results are means of six biological replicates with three technical replicate for each treatment and are presented as mean±SE. Different superscript (a,b,c,d) denotes significant differences among treatments (P ≤ 0.05).

**Supplementary Table 3: Differential expression of Ferritin1 gene in response to Fe/Zn stress *in Phaseolus vulgaris* L.**

| **Treatment** | **SHOOT** | | | | **ROOT** | | | |
| --- | --- | --- | --- | --- | --- | --- | --- | --- |
|  | **Mean Ct** | **ΔCt** | **ΔΔCt** | **2^-ΔΔCt^** | **Mean Ct** | **ΔCt** | **ΔΔCt** | **2^-ΔΔCt^** |
| **Control** | 20.649^a^±0.403 | 0.849^a^±0.403 | -1.087 | **1.000** | 19.822^a′^±0.359 | -2.178^a′^±0.359 | -6.333 | **1.000** |
| **0-Fe** | 21.529^a^±0.242 | 1.729^a^±0.242 | -0.207 | **0.543** | 26.155^b^′±0.205 | 4.155^b^′±0.205 | 0.000 | **0.012** |
| **0-Zn** | 20.583^a^±0.536 | 0.783^a^±0.536 | -1.153 | **1.046** | 23.296^c′^±0.242 | 1.296^c′^±0.242 | -2.859 | **0.090** |
| **300-Zn** | 21.736^a^±0.409 | 1.936^a^±0.409 | 0.000 | **0.470** | 23.430^c′^±0.230 | 1.430^c′^±0.230 | -2.725 | **0.082** |

The results are means of six biological replicates with three technical replicate for each treatment and are presented as mean±SE. Different superscript (a,b,c,d) denotes significant differences among treatments (P ≤ 0.05).

**Supplementary Table 4: Differential expression of FRO1 gene in response to Fe/Zn stress in *Phaseolus vulgaris* L.**

| **Treatment** | **SHOOT** | | | | **ROOT** | | | |
| --- | --- | --- | --- | --- | --- | --- | --- | --- |
|  | **Mean Ct** | **ΔCt** | **ΔΔCt** | **2^-ΔΔCt^** | **Mean Ct** | **ΔCt** | **ΔΔCt** | **2^-ΔΔCt^** |
| **Control** | 25.157^a^±0.233 | 5.357^a^±0.233 | -2.791 | **1.000** | 28.426^a′^±0.031 | 6.426^a′^±0.031 | 0.000 | **1.000** |
| **0-Fe** | 27.828^b^±0.374 | 8.028^b^±0.374 | -0.120 | **0.157** | 27.534^b′^±0.103 | 5.534^b′^±0.103 | -0.892 | **1.855** |
| **0-Zn** | 27.948^b^±0.462 | 8.148^b^±0.462 | 0.000 | **0.144** | 27.600^b′^±0.091 | 5.600^b′^±0.091 | -0.826 | **1.772** |
| **300-Zn** | 27.120^b^±0.238 | 7.320^b^±0.238 | -0.828 | **0.256** | 27.047^c′^±0.212 | 5.047^c′^±0.212 | -1.379 | **2.600** |

The results are means of six biological replicates with three technical replicate for each treatment and are presented as mean±SE. Different superscript (a,b,c,d) denotes significant differences among treatments (P ≤ 0.05).

**Supplementary Table 5: Differential expression of OPT3 gene in response to Fe/Zn stress in *Phaseolus vulgaris* L.**

| **Treatment** | **SHOOT** | | | | **ROOT** | | | |
| --- | --- | --- | --- | --- | --- | --- | --- | --- |
|  | **Mean Ct** | **ΔCt** | **ΔΔCt** | **2^-ΔΔCt^** | **Mean Ct** | **ΔCt** | **ΔΔCt** | **2^-ΔΔCt^** |
| **Control** | 19.451^a^±0.162 | -0.349^a^±0.162 | -0.077 | **1.000** | 22.006^a′^±0.261 | 0.006^a′^±0.261 | -3.518 | **1.000** |
| **0-Fe** | 18.957^ab^±0.182 | -0.843^ab^±0.182 | -0.571 | **1.408** | 20.325^b′^±0.290 | -1.675^b′^±0.290 | -5.199 | **3.208** |
| **0-Zn** | 19.528^a^±0.303 | -0.272^a^±0.303 | 0.000 | **0.948** | 25.524^c′^±0.232 | 3.524^c^±0.232 | 0.000 | **0.087** |
| **300-Zn** | 18.650^b^±0.129 | -1.150^b^±0.129 | -0.878 | **1.742** | 18.512^d′^±0.298 | -3.488^d′^±0.298 | -7.012 | **11.272** |

The results are means of six biological replicates with three technical replicate for each treatment and are presented as mean±SE. Different superscript (a,b,c,d) denotes significant differences among treatments (P ≤ 0.05).

**Supplementary Table 6: Differential expression of NRAMP1 gene in response to Fe/Zn stress *in Phaseolus vulgaris* L.**

| **Treatments** | **SHOOT** | | | | **ROOT** | | | |
| --- | --- | --- | --- | --- | --- | --- | --- | --- |
|  | **Mean Ct** | **ΔCt** | **ΔΔCt** | **2^-ΔΔCt^** | **Mean Ct** | **ΔCt** | **ΔΔCt** | **2^-ΔΔCt^** |
| **Control** | 22.548^a^±0.068 | 2.748^a^±0.068 | -0.85 | **1.000** | 23.224^a′^±0.252 | 1.224^a′^±0.252 | -1.994 | **1.000** |
| **0-Fe** | 22.609^a^±0.423 | 2.810^a^±0.423 | -0.788 | **0.957** | 23.315^a′^±0.204 | 1.315^a′^±0.204 | -1.903 | **0.938** |
| **0-Zn** | 22.981^a^±0.215 | 3.181^a^±0.215 | -0.417 | **0.740** | 25.218^b′^±0.164 | 3.218^b′^±0.164 | 0.000 | **0.251** |
| **300-Zn** | 23.398^a^±0.238 | 3.598^a^±0.238 | 0.000 | **0.554** | 21.971^c′^±0.252 | -0.029^c′^±0.252 | -3.247 | **2.383** |

The results are means of six biological replicates with three technical replicate for each treatment and are presented as mean±SE. Different superscript (a,b,c,d) denotes significant differences among treatments (P ≤ 0.05)

**Supplementary Table 7**: **Differential expression of NRAMP2 gene in response to Fe/Zn stress in *Phaseolus vulgaris* L.**

| **Treatments** | **SHOOT** | | | | **ROOT** | | | |
| --- | --- | --- | --- | --- | --- | --- | --- | --- |
|  | **Mean Ct** | **ΔCt** | **ΔΔCt** | **2^-ΔΔCt^** | **Mean Ct** | **ΔCt** | **ΔΔCt** | **2^-ΔΔCt^** |
| **Control** | 17.122^a^±0.166 | -2.678^a^±0.166 | -1.188 | **1.000** | 23.148^a′^±0.208 | 1.148^a′^±0.208 | -3.636 | **1.000** |
| **0-Fe** | 18.31^b^±0.153 | -1.49^b^±0.153 | 0.000 | **0.438** | 21.805^b′^±0.063 | -0.195^b′^±0.063 | -4.979 | **2.536** |
| **0-Zn** | 17.801^c^±0.073 | -1.999^c^±0.073 | -0.509 | **0.624** | 26.784^c′^±0.089 | 4.784^c′^±0.089 | 0.000 | **0.080** |
| **300-Zn** | 18.142^bc^±0.189 | -1.658^bc^±0.189 | -0.168 | **0.493** | 19.522^d′^±0.252 | -2.478^d′^±0.252 | -7.262 | **12.346** |

The results are means of six biological replicates with three technical replicate for each treatment and are presented as mean±SE. Different superscript (a,b,c,d) denotes significant differences among treatments (P ≤ 0.05)

**Supplementary Table 8: Differential expression of NRAMP3 gene in response to Fe/Zn stress in *Phaseolus vulgaris* L.**

| **Treatment** | **SHOOT** | | | | **ROOT** | | | |
| --- | --- | --- | --- | --- | --- | --- | --- | --- |
|  | **Mean Ct** | **ΔCt** | **ΔΔCt** | **2^-ΔΔCt^** | **Mean Ct** | **ΔCt** | **ΔΔCt** | **2^-ΔΔCt^** |
| **Control** | 18.985^a^±0.404 | -0.815^a^±0.404 | -0.371 | **1.000** | 23.238^a′^±0.150 | 1.238^a′^±0.150 | -2.362 | **1.000** |
| **0-Fe** | 19.356^a^±0.341 | -0.444^a^±0.341 | 0.000 | **0.773** | 22.300^b′^±0.232 | 0.300^b′^±0.232 | -3.300 | **1.916** |
| **0-Zn** | 19.356^a^±0.208 | -0.444^a^±0.208 | 0.000 | **0.773** | 25.600^c′^±0.258 | 3.600^c′^±0.258 | 0.000 | **0.195** |
| **300-Zn** | 19.300^a^±0.197 | -0.500^a^±0.197 | -0.056 | **0.803** | 20.235^d′^±0.147 | -1.765^d′^±0.147 | -5.365 | **8.017** |

The results are means of six biological replicates with three technical replicate for each treatment and are presented as mean±SE. Different superscript (a,b,c,d) denotes significant differences among treatments (P ≤ 0.05)

**Supplementary Table 9: Differential expression of ZIP2 gene in response to Fe/Zn stress in *Phaseolus vulgaris* L.**

| **Treatment** | **SHOOT** | | | | **ROOT** | | | |
| --- | --- | --- | --- | --- | --- | --- | --- | --- |
|  | **Mean Ct** | **ΔCt** | **ΔΔCt** | **2^-ΔΔCt^** | **Mean Ct** | **ΔCt** | **ΔΔCt** | **2^-ΔΔCt^** |
| **Control** | 24.557^ab^±0.292 | 4.757^ab^±0.292 | -0.102 | **1.000** | 24.437^a′^±0.343 | 2.437^a′^±0.343 | -1.748 | **1.000** |
| **0-Fe** | 23.491^b^±0.358 | 3.691^b^±0.358 | -1.168 | **2.093** | 24.470^a′^±0.351 | 2.470^a′^±0.351 | -1.715 | **0.977** |
| **0-Zn** | 24.659^a^±0.420 | 4.859^a^±0.420 | 0.000 | **0.931** | 26.185^b′^±0.395 | 4.185^b′^±0.395 | 0.000 | **0.297** |
| **300-Zn** | 24.621^a^±0.376 | 4.821^a^±0.376 | -0.038 | **0.956** | 22.907^c′^±0.250 | 0.907^c′^±0.250 | -3.278 | **2.888** |

The results are means of six biological replicates with three technical replicate for each treatment and are presented as mean±SE. Different superscript (a,b,c,d) denotes significant differences among treatments (P ≤ 0.05)

**Supplementary Table 10: Differential expression of HA2 gene in response to Fe/Zn stress in *Phaseolus vulgaris* L.**

| **Treatment** | **SHOOT** | | | | **ROOT** | | | |
| --- | --- | --- | --- | --- | --- | --- | --- | --- |
|  | **Mean Ct** | **ΔCt** | **ΔΔCt** | **2^-ΔΔCt^** | **Mean Ct** | **ΔCt** | **ΔΔCt** | **2^-ΔΔCt^** |
| **Control** | 19.504^a^±0.335 | -0.296^a^±0.335 | -0.147 | **1.000** | 19.417^a′^±0.125 | -2.583^a′^±0.125 | -2.67 | **1.000** |
| **0-Fe** | 19.086^a^±0.241 | -0.714^a^±0.241 | -0.565 | **1.336** | 20.288^b′^±0.064 | -1.712^b′^±0.064 | -1.799 | **0.546** |
| **0-Zn** | 19.651^a^±0.302 | -0.149^a^±0.302 | 0.000 | **0.903** | 22.087^c′^±0.205 | 0.087^c′^±0.205 | 0.000 | **0.157** |
| **300-Zn** | 18.835^a^±0.069 | -0.965^a^±0.069 | -0.816 | **1.589** | 18.238^d′^±0.143 | -3.762^d′^±0.143 | -3.849 | **2.264** |

The results are means of six biological replicates with three technical replicate for each treatment and are presented as mean±SE. Different superscript (a,b,c,d) denotes significant differences among treatments (P ≤ 0.05)

**Supplementary Table 11: Differential expression of GLP1 gene in response to Fe/Zn stress in *Phaseolus vulgaris* L.**

| **Treatment** | **SHOOT** | | | | **ROOT** | | | |
| --- | --- | --- | --- | --- | --- | --- | --- | --- |
|  | **Mean Ct** | **ΔCt** | **ΔΔCt** | **2^-ΔΔCt^** | **Mean Ct** | **ΔCt** | **ΔΔCt** | **2^-ΔΔCt^** |
| **Control** | 21.284^a^±0.125 | 1.484^a^±0.125 | -0.251 | **1.000** | 19.045^a′^±0.135 | -2.955^a′^±0.135 | -2.720 | **1.000** |
| **0-Fe** | 21.535^a^±0.178 | 1.735^a^±0.178 | 0.000 | **0.840** | 20.545^b′^±0.139 | -1.455^b′^±0.139 | -1.220 | **0.353** |
| **0-Zn** | 20.943^a^±0.104 | 1.143^a^±0.104 | -0.592 | **1.266** | 21.765^c′^±0.055 | -0.235^c′^±0.055 | 0.000 | **0.151** |
| **300-Zn** | 20.962^a^±0.122 | 1.162^a^±0.122 | -0.573 | **1.249** | 17.398^d′^±0.131 | -4.602^d′^±0.131 | -4.367 | **3.036** |

The results are means of six biological replicates with three technical replicate for each treatment and are presented as mean±SE. Different superscript (a,b,c,d) denotes significant differences among treatments (P ≤ 0.05)
